# *Tychonema* sp. BBK16 Characterisation: Lifestyle, Phylogeny and Related Phages

**DOI:** 10.3390/v15020442

**Published:** 2023-02-05

**Authors:** Peter Evseev, Irina Tikhonova, Andrei Krasnopeev, Ekaterina Sorokovikova, Anna Gladkikh, Oleg Timoshkin, Konstantin Miroshnikov, Olga Belykh

**Affiliations:** 1 Limnological Institute of the Siberian Branch of the Russian Academy of Sciences, 3 Ulan-Batorskaya Str., Irkutsk 664033, Russia; 2 Shemyakin-Ovchinnikov Institute of Bioorganic Chemistry of the Russian Academy of Sciences, 16/10 Miklukho-Maklaya Str., GSP-7, Moscow 117997, Russia; 3 Saint-Petersburg Pasteur Institute, 14 Mira Str., Saint-Petersburg 197101, Russia

**Keywords:** cyanobacterium, *Tychonema*, cyanopahges, proliferation of benthic cyanobacteria, freshwater ecology, mixotrophy, cyanobacterial genomics

## Abstract

Cyanobacterial expansion is harmful to the environment, the ecology of Lake Baikal and the economy of nearby regions and can be dangerous to people and animals. Since 2011, the process of colonisation of the lake with potentially toxic cyanobacteria belonging to the genus *Tychonema* has continued. An understanding of the mechanism of successful expansion of *Tychonema* requires scrutiny of biological and genomic features. *Tychonema* sp. BBK16 was isolated from the coastal zone of Lake Baikal. The morphology of BBK16 biofilm was studied with light, scanning electron and confocal microscopy. The biofilm is based on filaments of cyanobacteria, which are intertwined like felt; there are also dense fascicles of rope-like twisted filaments that impart heterogeneity to the surface of the biofilm. Genome sequencing, intergenomic comparisons and phylogenetic analyses indicated that *Tychonema* sp. BBK16 represent a new species related to planktic cyanobacterium *Tychonema bourrellyi*, isolated from Alpine lentic freshwater. Genome investigation revealed the genes possibly responsible for the mixotrophic lifestyle. The presence of CRISPR-Cas and restriction modification defence mechanisms allowed to suggest the existence of phages infecting *Tychonema* sp. BBK16. Analysis of CRISPR spacers and prophage-derived regions allowed to suggest related cyanophages. Genomic analysis supported the assumption that mobile elements and horizontal transfer participate in shaping the *Tychonema* sp. BBK16 genome. The findings of the current research suggest that the aptitude of *Tychonema* sp. BBK16 for biofilm formation and, possibly, its mixotrophic lifestyle provide adaptation advantages that lead to the successful expansion of this cyanobacterium in the Baikal’s conditions of freshwater lake environments.

## 1. Introduction

The global expansion of harmful cyanobacterial blooms in eutrophic waters constitutes a serious threat to freshwater ecology and public health [1,2]. The process of colonisation of Lake Baikal by cyanobacteria, initially named *Phormidium* spp., was first noticed in 2011 [3]. Further studies showed that this cyanobacterium belonged to the genus *Tychonema* (family Microcoleaceae, order Oscillatoriales), which was new to Baikal. The potentially toxic genus *Tychonema* proliferates in the benthos of Lake Baikal and now prevails in the biofilm ulcers of the Baikal sponge and other biofouling. The occupation of new ecological niches by these cyanobacteria raises questions about the biological and genetic mechanisms of such an evolutionary success.

Biofilm formation is an important evolutionary step in the colonisation of new ecological niches [4,5]. Cyanobacterial mats, which are laminated biofilms, are the oldest communities on Earth, dating back 3.5 billion years [6]. Although cyanobacterial mats formed by members of the genus *Tychonema* are rarely mentioned, it is known that they can cause problems, leading to animal poisoning with anatoxin, as in Lake Tegel and Reservoir Mandichosee, Germany, or the mass death of endemic sponges, as in Lake Baikal [3,7,8].

Bolshiye Koty is a small settlement on the coast of Lake Baikal, located within the territory of the Pribaikalsky National Park. It is fairly isolated, which facilitates nature preservation. There is no road, and the village can only be reached by water or on foot. The settlement is located on a popular tourist route—the Great Baikal Trail. Since 2009, the coastal zone of Bolshiye Koty has been included in interdisciplinary ecological studies of the splash zone of Lake Baikal in order to study the natural course of hydrobiological processes in this area, including the composition of cyanobacteria [9]. The appearance of biofilms uncharacteristic of Lake Baikal on the surface of stones and wooden substrates in this area has been noted since 2010 [3].

Cyanobacteria are characterised by the ability to carry out oxygenic photosynthesis [10], but the capability of mixotrophy has also been reported [11,12]. Mixotrophy has traditionally been defined as an alternative form of carbon uptake but may also involve the acquisition of molecules containing nitrogen, phosphorus, trace minerals, vitamins and high-energy compounds such as ATP. *Sensu lato* mixotrophy can be considered a combination of different nutritional pathways in one organism [12,13]. The capacity for mixotrophy provides a significant competitive advantage, enabling mixotrophic bacteria to dominate broader aquatic environments since they can utilise more resources than photoautotrophic bacteria [14].

It is important that Lake Baikal is characterised by its oligotrophy [15]. However, the concentration of total organic carbon (C_org_) in bottom water in the coastal zone of Lake Baikal is 1.5–2.0 mg C/L, and the concentration of total organic nitrogen is 0.33–0.47 mg/L [16], exceeding such values in the oligotrophic pelagial zone of the lake by two times. In biofilms, the content of C_org_ is much higher due to the activity of photoautotrophic organisms (cyanobacteria, algae and macrophytes) and chemoautotrophic bacteria capable of synthesising organic carbon. Thus, in biofilms taken near the Bolshiye Koty settlement, the C_org_ was found to be 35 mg/L, total phosphorus to be 0.022 mg/L and total nitrogen 0.154 mg/L. [16]. In the bottom water at the same station, the concentration of total carbon was 5.6 mg/L, total phosphorus 0.007 mg/L and total nitrogen 0.142 mg/L. These conditions may contribute to the evolutionary success of mixotrophic cyanobacteria.

It is possible to suggest that Baikal cyanobacteria might also use the advantages of mixotrophy to expand their ecological niche. The ability to uptake organic compounds is related to the presence of transport proteins, and genes genomic analysis of various cyanobacteria, including marine cyanobacteria *Prochlorococcus* and *Synechococcus*, has demonstrated the presence of genes encoding amino acid, phosphate and sugar transporters [12,17,18,19]. It would be interesting to check for the presence of mixotrophy-related genes in the genomes of Baikal cyanobacteria and related ones.

The environmental adaptation of bacteria, in particular, is affected by bacteriophages (aka phages) [20], viruses that infect bacteria. Cyanobacterial phages (cyanophages) contribute to the evolution of cyanobacteria and can be considered a reservoir of genes important for the ecological adaptation of cyanobacteria [21]. These genes can alter the physiology of an infected cyanobacterium, increasing the production of progeny phage [22]. At the beginning of 2022, the NCBI Genome database contained about 500 sequences of complete genomes of cyanophages, most of which were isolated from marine *Synechococcus* and *Prochlorococcus* (order Synechococcales), but there are only a few examples of isolated and published cyanophages infecting Oscillatoriales cyanobacteria [23,24,25,26,27,28,29], and no *Tychonema* phages are described to date. However, some information about phage–host interactions can be recovered using the analysis of genomic CRISPR arrays [30,31,32]. Moreover, temperate phages that insert their genome into the host chromosome can leave traces of phage infection [33,34,35,36].

The purpose of this study was to analyse the biofilm formed by the benthic *Tychonema* sp. strain BBK16 isolated in the coastal zone of Lake Baikal and to characterise the general genomics of the cyanobacterium, including the analysis of CRISPR spacers and prophage-derived regions. Another intention of this research was to investigate the possibility of the mixotrophy of *Tychonema* sp. BBK16, using bioinformatic analysis, to reveal the mechanisms that can assist the expansion of *Tychonema* in the lake.

## 2. Materials and Methods

### 2.1. Sampling Location

**Figure 1:**
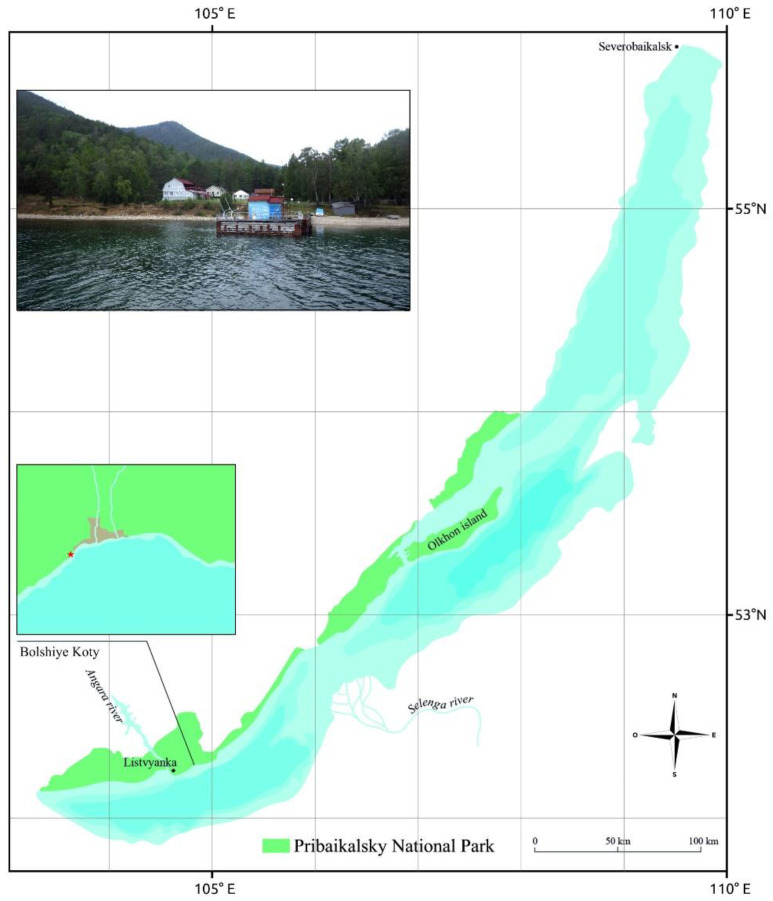
Map of Lake Baikal, Pribaikalsky National Park, and Bolshiye Koty, from where samples were obtained.

Biofilm samples were obtained in 2015–2016 from the underwater part of the wooden pier located in the area of the Scientific Research Station “Bolshiye Koty” of the Limnological Institute (Bolshiye Koty Settlement, 51.883333° N, 105.05° E) (Figure 1). Native biofilm macro photography was carried out using a Pentax WG-3 GPS camera (Ricoh Imaging, Tokyo, Japan).

### 2.2. Cultivation and Biofilm Visual Analysis

During cultivation, biofilm fragments were washed with sterile Z-8 mineral medium [37], crushed into smaller pieces and placed on agar plates with the same medium in an incubator. The cultivation conditions were as follows: illumination 1200 lux, the light mode consisted of alternating night and day periods 16:8, temperature 11–12 °C. Next, individual trichomes were sterilely excised from agar, suspended in a liquid medium and cultured again to obtain a unialgal culture.

Microphotographs of *Tychonema* sp. BBK16 were obtained using an Axio Imager light microscope (Carl Zeiss, Jena, Germany). The structure of the biofilm surface was visualised using scanning electron microscopy (SEM). First, thin sterile glasses were introduced into the liquid medium with cyanobacteria, which, after fouling, were fixed with 2% formaldehyde and dehydrated in an ethanol concentration gradient. Subsequently, the glasses with *Tychonema* biofouling were dried at 40 °C, coated in gold using a Balzers SCD 004 sputter-coater (Bal-Tec AG, Balzers, Liechtenstein) and examined using SEM Quanta 200 (FEI Co., Hillsboro, OR, USA). Scanning laser confocal microscopy studies were carried out on an LSM 710 microscope (Carl Zeiss) equipped with a helium–neon laser (561 nm), which causes the autofluorescence of cyanobacterial pigments. Biofilm images were obtained using ZEN 2010 software (Carl Zeiss), and 3D reconstruction was achieved with Imaris software (Bitplane Scientific Software, Zürich, Switzerland).

### 2.3. DNA Extraction and Sequencing

The total DNA was extracted by enzymatic lysis using lysozyme (Roche, Basel, Switzerland), proteinase K (Thermo Scientific, Waltham, MA, USA) and sodium dodecyl sulphate (VWR Life Science, Radnor, PA, USA), followed by phenol and chloroform (Medigen, Novosibirsk, Russia) extraction [38]. The NEBNextUltra DNA library prep kit for Illumina (New England BioLabs, Ipswich, MA, USA) was used for DNA library construction. DNA samples were sequenced to generate 300 bp paired-end reads using the Illumina MiSeq platform. De novo genome assembly was performed using SPAdes 3.12 [39], binning was conducted using MaxBin 2.0 [40], and the assembly was manually curated using a BLASTN [41] search with default settings against the NCBI Bacterial GenBank Database [42]. The resulting genome was deposited in GenBank (Accession # JAKJHX000000000).

### 2.4. Genome Annotation and Prediction of Gene Functions

The assembled genome was annotated using the Prokaryotic Genome Annotation Pipeline (PGAP) [43] with default settings. Additionally, annotations of all genomic regions shown in this study were manually curated. Manual curation included checking the positions of open reading frames (ORFs) and functional assignment. Checking the ORF positions was conducted using Geneious Prime 2022.0.1 tools (Biomatters, Inc., Auckland, New Zealand) [44] and Gimmer 3.0.2 [45]. Gene functional assignment was performed using the BLAST search with nr/nt database and HMM-HMM motif comparison. The parameters of BLAST search were: BLASTP algorithm, E-value < 1 × 10^−5^ and other parameters were default. The HMM-HMM motif comparison was performed using the HHpred server [46] and PDB, SCOPe, CATH and UniProt-SwissProt-viral databases. Pfam domains were identified using pfam_scan 1.6 tool [47] applying default settings. Clusters of orthologous groups of proteins (COGs) were identified using the eggNOG-mapper 2 server [48] applying the “Genomic” settings.

### 2.5. Average Nucleotide Identity Calculations and Phylogenetic Analysis

Cyanobacterial genome and gene sequences were downloaded from the NCBI GenBank. The relevance of species names was checked in Algaebase (https://www.algaebase.org, accessed on 10 January 2023). The average nucleotide identity (ANI) matrix was calculated using ANI/AAI-Matrix Genome-based distance matrix calculator (Kostas lab, Atlanta, GA, USA) and Bio-NJ clustering [49]. Alignments of 16S rDNA sequences and protein sequences were performed with MAFFT 7.48 [50] with default settings and using the L-INS-i algorithm. 16S rDNA and single protein phylogenetic trees were constructed using RAxML-NG 1.1.0 [51] and the raxmlGUI 2.0.10 graphic interface [52] with (--tree rand{10} --bs-trees 1000) settings and applying the best protein model found with ModelTest-NG 0.1.7 [53]. The robustness of the RAxML-NG 1.1.0 trees was assessed using bootstrapping and calculations of transfer bootstrap estimation (TBE) support [54].

Alignment of concatenated sequences of orthologous proteins was obtained with PhyloPhlAn 3.0 applying (-d phylophlan-diversity medium -f supermatrix_aa.cfg) settings. The tree was constructed using RAxML-NG 1.1.0 with (-tree rand{1}-bs-trees 100) settings. Other details of the phylogenetic analysis using concatenated alignments were the same as described above.

### 2.6. Identification of CRISPR Loci and Prophage-Derived Regions

CRISPR loci were identified with MinCED [55] using “-gffFull” settings. Spacer sequences were extracted with MinCED using the “-spacers” settings. Prophage-derived regions were found using the PHASTER server [56] applying default settings. Similarity of genes of supposedly prophage origin was estimated using built-in PHASTER utilities.

## 3. Results

### 3.1. Biofilm Characterisation

**Figure 2:**
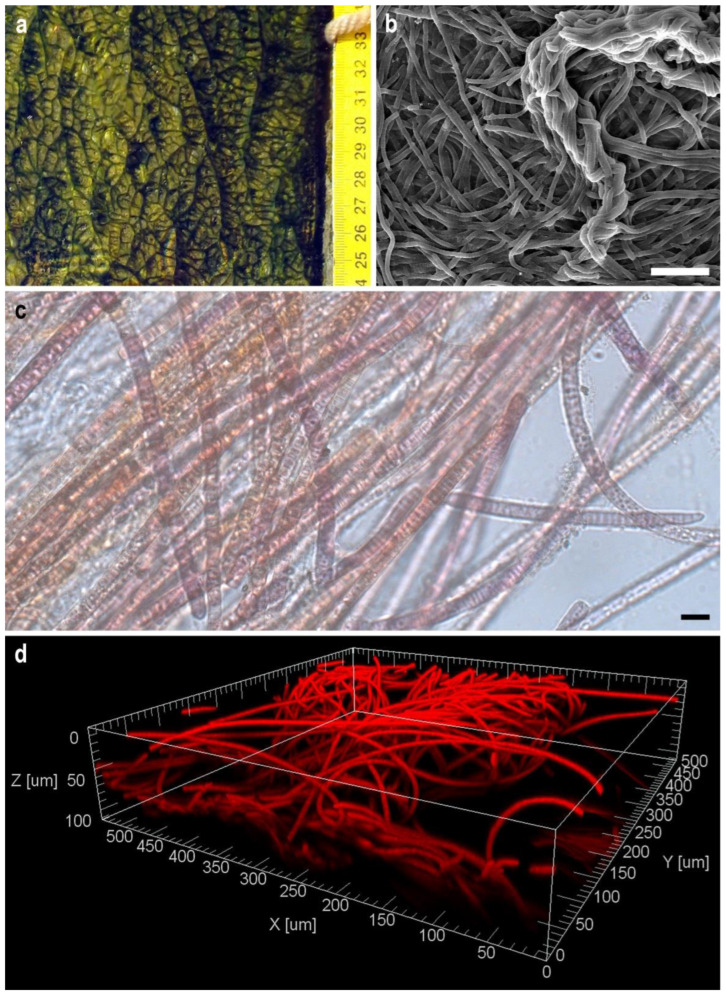
Macro photography of a natural biofilm from the fouling of the pier, the source of isolation of the strain *Tychonema* sp. BBK16 (**a**). Morphology of the biofilm of *Tychonema* sp. BBK16 formed in culture: (**b**) scanning electron microscopy. Scale bar = 50 µm; (**c**) light microscopy. Scale bar =10 µm; (**d**) confocal microscopy.

The fouling that was the source of strain isolation was a dense, leathery, olive-green biofilm covering the underwater surface of the wooden pier (Figure 2a). SEM visualisation of the structure of a biofilm formed by *Tychonema* sp. BBK16 in a liquid medium showed that it was based on cyanobacterial filaments intertwined like felt and the presence of dense fascicles of rope-like twisted filaments that imparted heterogeneity to the biofilm surface, as observed in nature (Figure 2b). Light microscopy showed that the trichomes of the strain were straight and that they were enclosed in thin, transparent polysaccharide sheaths, which strengthened the structure of the biofilm (Figure 2c). Since the sheaths were thin, the cells were clearly visible, even in SEM (Figure 2b). The autofluorescence of chlorophyll and the phycobiliproteins of cyanobacteria enabled visualisation of the 3D structure of the biofilm using laser scanning microscopy without additional staining (Figure 2d). Confocal microscopy showed that the biofilm consisted of randomly intertwined threads and had a multilayer structure.

### 3.2. General Genomic Features and Intergenomic Comparisons

The total size of the *Tychonema* sp. BBK16 draft genome is 5,267,730 nucleotides (nt), which is close to the size of the genome of *Tychonema bourrellyi* FEM_GT703 (5,081,867 nt) isolated from a freshwater sample taken from Lake Garda [57] and less than the genome size of other *Tychonema* genomes contained in the NCBI GenBank Database (Table 1). The GC-content of 44.3% is close to the GC-content of other *Tychonema* genomes and is slightly closer to the GC-content of *T. bourrellyi* FEM_GT703 (44.7%) (Table 1). All *Tychonema* genomes were deposited as draft sequences.

The average nucleotide identity (ANI) calculations were performed using all available genomes of *Tychonema* and other related cyanobacterial strains found with BLAST search using BBK16 rDNA sequences. The maximum ANI value of about 91% was found for the *Tychonema* sp. BBK16–*T. bourrellyi* FEM_GT703 pair (Supplementary Figure S1). This value is lower than the 95–96% ANI cutoff, the standard most frequently used for prokaryotic species demarcation using complete or nearly complete genomes [58,59,60]. The next highest ANI values of about 83% corresponded to other *Tychonema* strains and *Microcoleus* sp. LEGE 07076. This value is higher than the estimated prokaryotic mean demarcation boundaries of the genus, which is about 74% [61].

### 3.3. Phylogenetic Analyses

**Figure 3:**
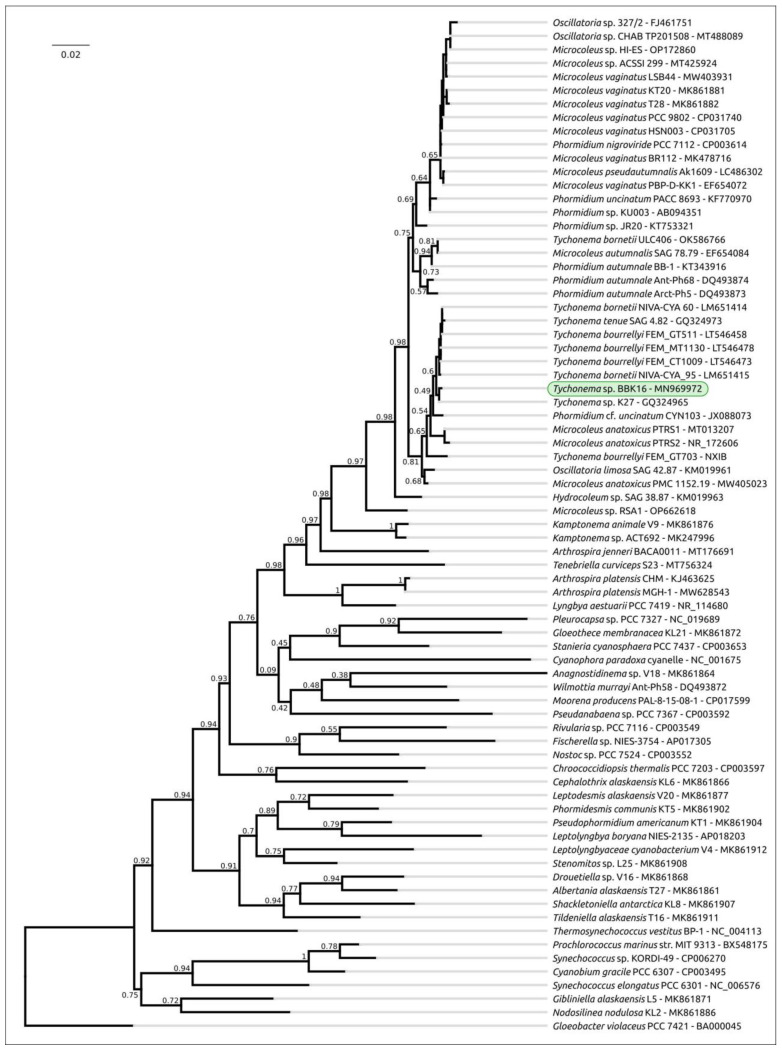
Best-scoring ML phylogenetic tree constructed with 75 nucleotide sequences of 16S rDNA. The NCBI accession number is shown to the right of the organism’s name. *Gloeobacter violaceus* PCC 7421 was used as an outgroup. The numbers near the tree branches indicate the TBE values. The total number of bootstrap trees was 1000. The scale bar shows 0.02 estimated substitutions per site.

**Figure 4:**
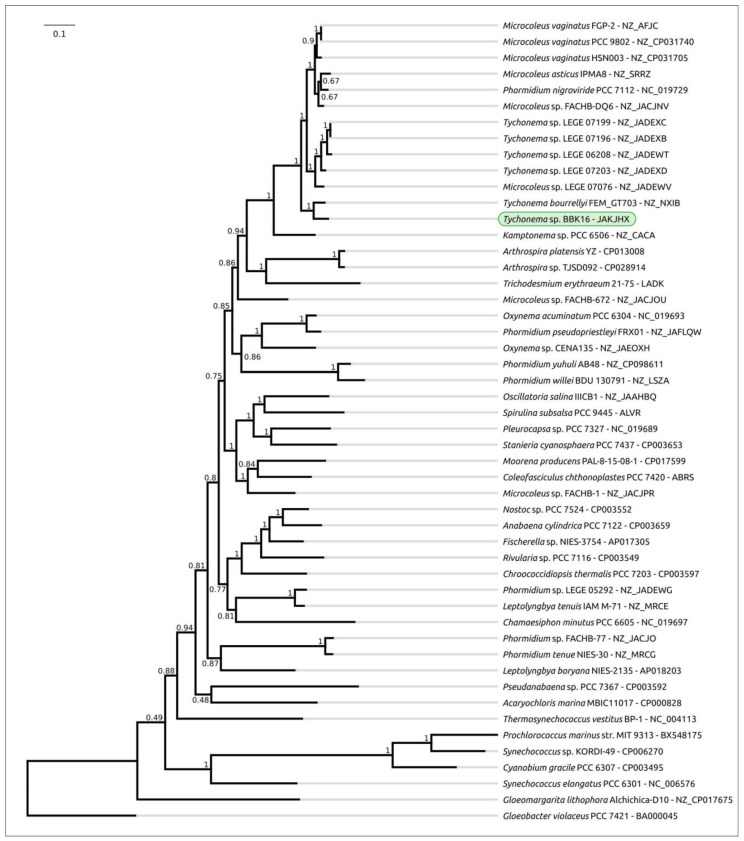
Best-scoring ML phylogenetic tree constructed with 50 concatenated amino acid sequences of conserved proteins found with PhyloPhlAn. The NCBI accession is shown to the right of the organism’s name. *Gloeobacter violaceus* PCC 7421 was used as an outgroup. The numbers near the tree branches indicate the TBE values. The total number of bootstrap trees was 100. The scale bar shows 0.1 estimated substitutions per site.

Phylogenetic analysis was performed using 16S rDNA and concatenated alignments of orthologous proteins. The 16S rDNA representative sequences were selected using the most similar sequences found with the BLAST search and NCBI nt databases using the sequences of Oscillatoriales species mentioned in [62] and sequences of more distantly related organisms used in [63]. The 16S rDNA phylogenetic tree (Figure 3) places all the *Tychonema* strains and several *Microcoleus*, *Phormidium* and *Oscillatoria* strains into a clade with a TBE support of 0.81. Phylogenetic analysis using the PhyloPhlAn pipeline, which employs 400 most conserved proteins, can show better resolution and higher bootstrap support. The PhyloPhlAn phylogenetic tree (Figure 4) using the sequences representing most of the available *Tychonema*/*Microcoleus*/*Phormidium* full genomic sequences and other cyanobacterial groups placed *Tychonema* sp. BBK16 and *T. bourrellyi* FEM_GT703 into a distinct clade and depicted a group of *Tychonema* strains as a paraphyletic group.

### 3.4. General Proteome Features

**Figure 5:**
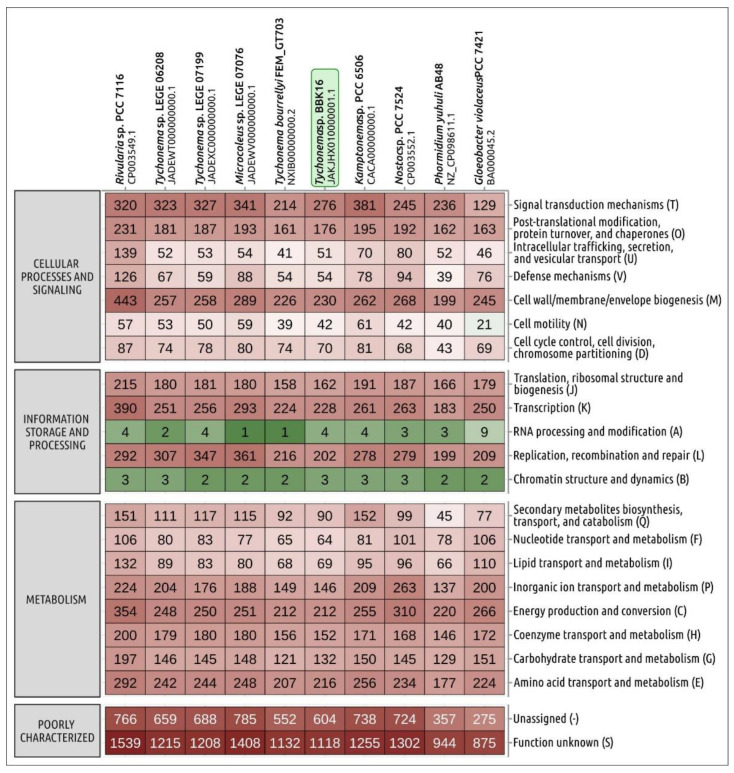
Heatmap showing the distribution of clusters of orthologous groups of proteins of *Tychonema* sp. BBK16 and other cyanobacteria obtained using eggNOG-mapper. The numbers displayed in cells indicate the number of proteins belonging to the orthologous groups.

The *Tychonema* strains deposited in GenBank clearly exhibit diversity in genome size and the number of predicted proteins (Table 1). There are 4708 protein-coding sequences predicted in the BBK16 genome, which is close to the number of proteins encoded in the FEM_GT703 genome (4629) and less than in other *Tychonema* genomes. Comparison of the distribution of clusters of orthologous groups of proteins (COGs) of *Tychonema* and several other cyanobacterial strains indicated that the number of orthologous groups assigned to categories V (defence mechanisms), N (cell motility), L (replication, recombination and repair), K (transcription) and to several other categories in *Tychonema* sp. BBK16 was similar to that in *T. bourrellyi* FEM_GT703, seemingly indicating the relatedness of these species (Figure 5).

Transposases are of particular importance for bacteria. In freshwater, cyanobacteria provide the basis for rapid adaptation and survival in harsh freshwater environments [64]. The *Tychonema* sp. BBK16 genome content analysis also indicated the presence of 29 transposase-encoding genes, which is somewhat lower than in genomes of other analysed *Tychonema* strains (32–42 genes) and noticeably lower than in *Microcoleus* sp. LEGE 07076 (52 genes) (Table 2). Interestingly, a similar situation is observed for restriction modification enzymes, which are important for protecting the cell from phages [65,66]. The *Tychonema* sp. BBK16 genome contains 62 genes of restriction modification enzymes, while other analysed *Tychonema* strains have 81–110 genes, and *Microcoleus* sp. LEGE 07076 has 110 such genes (Table 2).

### 3.5. Mixotrophy-Associated Proteins

The ability to assimilate organic nutrients detected in both marine and freshwater cyanobacteria is related to transporters needed for the uptake of organic compounds [12,67]. Pfam domain search predicted that the genome of *Tychonema* sp. BBK16 contains four genes to encode the proteins containing sugar-transporter-like domains. Experimentally, the uptake of sugars in cyanobacteria has been shown to be associated with the presence of GlcH permease importer, a high-affinity glucose transporter (called “Pro1404” in *Prochlorococcus marinus* SS120, where it was first studied) [68,69,70] and GlcP permease [71,72].

The BLAST search conducted using the Pro1404 amino acid sequence, NCBI nr database and 50 cyanobacterial genomes used in phylogenetic analysis (Figure 4) indicated the presence of genes encoding GlcH in all 50 genomes mentioned above and in numerous other cyanobacteria (E-value < 1 × 10^−5^). Some of the 50 genomes contained two or three copies of the *glcH*-like gene. These results are consistent with a result published earlier using another dataset [12]. The genomes of all the analysed strains of *Tychonema*, *Microcoleus* and *Kamptonema* carry a single copy of the *glcH*-like gene.

Phylogenetic analysis using GlcH-like amino cyanobacterial acid and sequences of the closest homologues from other bacterial taxa indicate the complex evolutionary history of this protein (Supplementary Figure S2). The large clade containing Pro1404 also includes sequences from noncyanobacterial taxa, and some strains appear to carry genes resulting from duplication events.

**Figure 6:**
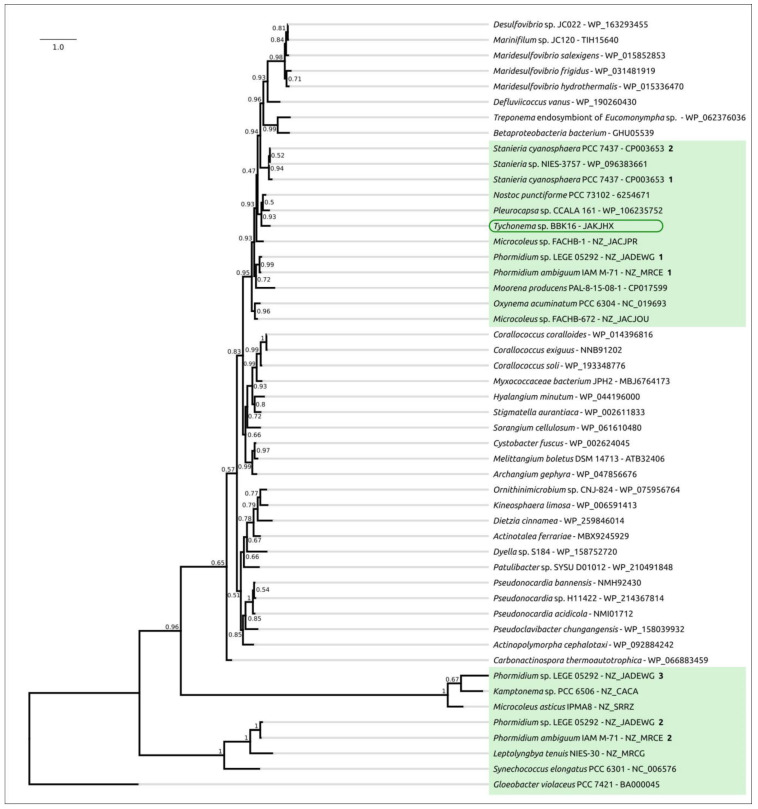
Best-scoring ML phylogenetic tree constructed with 50 amino acid sequences of GlcP protein. The NCBI accession number is shown to the right of the organism’s name. *Gloeobacter violaceus* PCC 7421 was used as an outgroup. The numbers near the tree branches indicate the TBE values. Bold numbers to the right of NCBI accession indicate the multiple instances of encoded proteins. The total number of bootstrap trees was 1000. The scale bar shows 1.0 estimated substitutions per site.

Glucose:H+ symporters GlcP were searched in a similar way but using a sequence of experimentally studied permease from *Nostoc punctiforme* [72,73]. In contrast, genes encoding GlcP-like proteins have only been found in 14 of the 50 genomes mentioned above. Interestingly, no genomes of *Tychonema*, with the exception of BBK16, carry the *glcP* gene. Phylogenetic analysis indicated, with high bootstrap support (TBE 0.93), a close relatedness of *Tychonema* sp. BBK16, *Microcoleus* sp. FACHB-1, *Nostoc punctiforme* PCC 73102 and *Pleurocapsa* sp. CCALA 161 proteins (Figure 6). The phylogeny of GlcP may indicate multiple events of horizontal transfer accompanying the evolution of GlcP, which is consistent with a result published earlier [72].

The genome of *Tychonema* sp. BBK16 encodes multiple amino acid ABC transporters. These are proteins that can participate in the import of amino acids, which can be a source of carbon and nitrogen [12]. A homology search revealed that most of the transporters discussed in meticulous work on mixotrophy [12], probably involved in the uptake of amino acids and other organic compounds, are encoded in the genome of BBK16 and other *Tychonema* strains. The genome contains the *aapJQMP* locus encoding the amino acid transport system (contig JAKJHX010000121.1), the *phnECD* locus encoding the phosphonate transport system (contig JAKJHX010000165.1), homologues of *glnQ*, the gene encoding glutamine transport system ATP-binding protein and homologues of *proV* and *proW* genes encoding the transporters participating in the uptake of dimethylsulfoniopropionate (DMSP).

### 3.6. Analysis of CRISPR Loci

**Figure 7:**
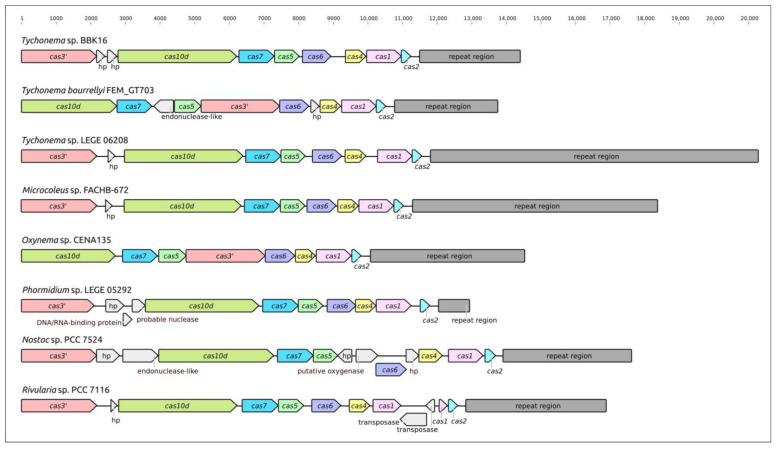
Genetic map of the CRISPR loci of *Tychonema* sp. BBK16 and other cyanobacteria showing the location of *cas* gene, adjacent genes and spacer arrays (repeat regions). Sequences designated “hp” correspond to genes encoding hypothetical proteins. The scale above the sequences indicates the position in the locus.

Analysis of *Tychonema* sp. BBK16 and other cyanobacterial genomes revealed the presence of CRISPR-Cas phage defence systems (Figure 7). The analysed systems belong to class 1, which encodes multisubunit effector complexes. The organisation of loci indicated its belonging to type I and subtype I-D, typical of cyanobacteria [74,75]. The sequence search indicated that most *Tychonema* Cas proteins have close homologues in other cyanobacteria. Interestingly, the *Tychonema* sp. BBK16 CRISPR locus contains two hypothetical protein genes downstream of the *cas3′* gene. Other analysed cyanobacterial genomes CRISPR loci may also contain additional inserted genes, including transposases. In some genomes, the order of the *cas* genes may be different, presumably due to recombinations. The size of the *Tychonema* sp. BBK16 CRISPR arrays (2906 bp) is similar to that of *Tychonema bourrellyi* FEM_GT703 (2978 bp).

The CRISPR array of *Tychonema* sp. BBK16 contains 39 spacers. The BLAST search using NCBI GenBank Phage database indicated similarities of spacer sequences with genomic sequences of several dozens of isolated cyanophages, but there were no exact matches found. According to the results of the search, 8 spacers demonstrated higher similarity to cyanophages, and 31 spacers were more similar to other phages (Table 3).

### 3.7. Search for Prophage-Derived Regions

**Figure 8:**
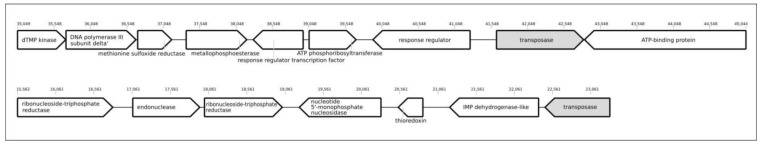
Genetic map of suggested prophage-derived regions predicted by PHASTER in genome contigs of *Tychonema* sp. The scale above the sequence indicates the position in the corresponding contig.

Search for prophages-derived regions (PDRs) in the genome of *Tychonema* sp. BBK16, performed with PHASTER, revealed two regions (Figure 8). Examination of genome content indicated the absence of structural proteins and the presence of transposases in both PDRs. Homologous genes were found in *Nodularia* phage vB_NspS-kac65v151 (NCBI accession NC_048756, genus *Ravarandavirus*), *Synechococcus* phage S-CBP2 (NC_025455, family *Autographiviridae*), *Synechococcus* phage S-CBS2 (NC_015463, unclassified siphovirus) and other phages. The presence of transposase can indicate genetic exchanges involving phage and host genomes.

### 3.8. Transporter Genes in Phage Genomes

To check the assumption of the involvement of bacteriophages in the transmission of transporter genes, including genes that may be useful for a mixotrophic lifestyle, the *Tychonema* sp. BBK16 mixotrophy-associated proteins were used for searching the homologous sequences in phages using the NCBI nt database. In addition, all GenBank phage database sequences (about 33 thousand sequences as of January 2023) were checked for the presence of transporter annotations.

The *glcH* and *glcP* genes were not found in the genomes of isolated cyanophage, but homologues GlcH and GlcP were found to be encoded in several phage human metagenomic sequences, including sequences MK231526.1 and BK032514.1. ABC transporter ATP-binding protein genes probably related to *glnQ* were found in the complete genome of isolated freshwater temperate cyanophage *Planktothrix* phage PaV-LD [27] and partial genomic sequences of another temperate freshwater cyanophage AS-1 [76]. Interestingly, sequences with a pairwise identity of 97% and above with the *Planktothrix* phage PaV-LD ATP-binding protein gene were found in several *Planktothrix* cyanobacterial genomes (Supplementary Figure S3), indicating that *Planktothrix* phage PaV-LD can represent a group of related temperate phages. Twenty other complete GenBank genomes of tailed cyanophages contain the genes annotated as ABC transporters, basically encoding the phosphate ABC transporter. A total of 14,353 complete genomes of *Caudoviricetes* phages deposited in the NCBI Genome database contain 603 genes annotated as different genes of the ABC transporter protein.

## 4. Discussion

Benthic biofilms are highly adapted to changing environmental conditions (dramatic changes in environmental parameters and nutrient concentrations) and are absorbers of excess nutrients from the environment [77]. The trichomes of the Baikal strain tightly intertwine and form a biofilm, characteristic of many species of Oscillatoriales cyanobacteria, in which the bacteria are held together by a polysaccharide mucus that is able to withstand harsh environmental conditions that might involve nutrient deficiencies and physical stress [78]. Various types of microscopy have been used to study the morphology of biofilms. SEM enables visualisation of the surface of the film, as well as its layers in cross-section, and confocal microscopy, which examines the film’s internal structure [79,80]. The structure of the *Tychonema* BBK16 biofilm was very similar to the 3D structures formed by its closest relatives, cyanobacteria of genera *Microcoleus* and *Tychonema*. *Microcoleus* and *Tychonema* form microbial mats in the benthos of lakes and rivers [77,81].

The genomic analysis showed a similar genome size and GC-content of *Tychonema* sp. BBK16 and *Tychonema bourrellyi* FEM_GT703. Calculations of ANI using all available *Tychonema* genomes and genomes of related cyanobacterial strains indicated that *Tychonema* sp. BBK16 may be classified as a representative of a new species. Phylogenetic analysis using concatenated alignments of orthologous protein sequences placed *Tychonema* sp. BBK16 and *T. bourrellyi* FEM_GT703 in a distinct clade and showed a complex picture of taxonomic relations between the strains classified as belonging to the Microcoleaceae family of the Oscillatoriales order. The proteome analysis also indicated close relatedness of *Tychonema* sp. BBK16 and *T. bourrellyi* FEM_GT703.

Previously, representatives of the genus *Tychonema* capable of forming biofilms were described in karst streams in a community with heterotrophic bacteria [82]. It has been shown that during the destruction of organic substances at the bottom of streams, a significant amount of organic matter is released, which is consumed by cyanobacteria in the lower layers of biofilms, where cells far from light are not able to photosynthesise. The bioinformatic analysis of the *Tychonema* sp. BBK16 genome indicated the presence of genes that can participate in the import of organic compounds. One of the sugar uptake genes, *glcP*, could be obtained by horizontal transfer. The *Tychonema* sp. BBK16 genome also contains clusters of genes of the *aapJQMP* amino acid transport system, the *phnECD* phosphonate transport system and other nutrient import genes. We hypothesise that cyanobacterial mixotrophy may be a factor for survival for *Tychonema* sp. BBK16 in benthic biofilms. It can be assumed that cyanobacteria of this genus are well adapted to the harsh conditions of Lake Baikal. The presence of protective polysaccharide sheaths, the ability to form dense biofilms and, probably, the capability of mixotrophy helped this species to occupy different ecological niches within the lake. We can also speculate that some genes associated with mixotrophy may be transferred by phages or other mobile elements.

It seems that there are no published isolated *Tychonema* phages at the moment. However, bioinformatic analysis indicated the presence of CRISPR-Cas and restriction modification phage defence systems in *Tychonema* sp. BBK16 genome. The *Tychonema* sp. BBK16 genome CRISPR-Cas locus belongs to characteristics for cyanobacteria subtype I-D of class 1 and includes a CRISPR array containing 39 spacers. Search for similar phages using the *Tychonema* sp. BBK16 spacers found no related cyanophages for about 80% of spacers, which can be related to the lack of genomic data on cyanophages [32]. Isolated cyanophages exhibiting similarities in genomic sequences with the CRISPR spacers of *Tychonema* sp. BBK16 belong to *Kyanoviridae*, *Tamkungvirus* and other phages.

Prophage predictions indicated the presence of prophage remnants in the *Tychonema* sp. BBK16 genome. These regions could be transferred in the genomes with the participation of transposases. Prophage-derived regions contain genes resembling cyanophage homologues in the genomes of *Nodularia* phage vB_NspS-kac65v151, *Synechococcus* phage S-CBP2 and *Synechococcus* phage S-CBS2.

## 5. Conclusions

Cyanobacterium *Tychonema* sp. BBK16 was isolated from the benthic zone of Lake Baikal. In culture, the strain formed biofilms with a felt-like interweaving of trichomes. Long fascicles of twisted trichomes were observed on the surface of the film, which caused folding. The ability to synthesise polysaccharide sheaths enabled the formation of more durable biofilms. Confocal microscopy showed the layered structure of the *Tychonema* biofilm. The genome of BBK16 was sequenced and analysed. The results of intergenomic comparisons and phylogenetic analyses indicate that *Tychonema* sp. BBK16 represent a new species related to *Tychonema bourrellyi* strain FEM_GT703. Genomic analysis indicated the presence of mixotrophy-associated genes. Analysis of CRISPR spacers and prophage-derived regions revealed cyanophages possibly related to hypothetical *Tychonema* phages.

## Figures and Tables

**Table 1:** General features of *Tychonema* genomes.

Organism	NCBI Accession	# Nucleotides	# Sequences	GC-Content, %	# CDS
*Tychonema bourrellyi* FEM_GT703	NZ_NXIB00000000.2	5,081,867	271	44.7	4629
*Tychonema* sp. BBK16	NZ_JAKJHX000000000.1	5,267,730	226	44.3	4708
*Tychonema* sp. LEGE 06208	NZ_JADEWT000000000.1	6,554,502	233	45.7	5667
*Tychonema* sp. LEGE 07196	NZ_JADEXB000000000.1	6,690,183	226	45.6	5658
*Tychonema* sp. LEGE 07199	NZ_JADEXC000000000.1	6,699,634	343	45.7	5688
*Tychonema* sp. LEGE 07203	NZ_JADEXD000000000.1	6,618,298	351	46.0	5800

**Table 2:** Number of genes of transposes and restriction modification enzymes in cyanobacterial genomes predicted using eggNOG-mapper 2.

Name	Number of Restriction Modification Enzyme Genes	Number of Transposase Genes
*Tychonema* sp. BBK16	62	27
*Tychonema bourrellyi* FEM_GT703	82	38
*Tychonema* sp. LEGE 06208	77	42
*Tychonema* sp. LEGE 07199	81	32
*Microcoleus* sp. LEGE 07076	110	50
*Kamptonema* sp. PCC 6506	65	34
*Nostoc* sp. PCC 7524	61	25
*Phormidium yuhuli* AB48	54	27
*Rivularia* sp. PCC 7116	45	39
*Gloeobacter violaceus* PCC 7421	42	18

**Table 3:** Cyanophages demonstrating sequence similarities with CRISPR spacers found with the BLAST search (the searches’ first hits).

Spacer	Phage	Taxonomy	Accession Number	Identical Sites, %	Pairwise Identity, %	Query Coverage, %
4	Cyanophage P-RSM6	*Kyanoviridae; Sokavirus*	HQ634193.1	95.0	95.0	48.78
8	*Synechococcus* phage S-T4	*Tamkungvirus*	MH412654.1	88.0	88.0	73.53
9	*Synechococcus* phage S-SRM01	Unclassified myovirus	MW015081.1	100	100	57.58
19	*Synechococcus* phage S-T4	*Tamkungvirus*	MH412654.1	91.7	91.7	68.57
20	*Synechococcus* phage B3	Unclassified myovirus	MN695334.1	100	100	48.72
23	*Synechococcus* phage S-RIM2	*Kyanoviridae; Nerrivikvirus*	KX349226.1	91.3	91.3	69.70
29	*Synechococcus* phage S-SM1	*Kyanoviridae; Thetisvirus*	GU071094.1	100	100	48.57
37	*Synechococcus* phage S-H68	Unclassified myovirus	MK016663.1	95.5	95.5	45.83

## Data Availability

Not applicable.

## Supplementary Materials

The following are available online at https://www.mdpi.com/article/10.3390/v15020442/s1, Figure S1: ANI matrix constructed with cyanobacterial 50 genomes using ANI/AAI-Matrix Genome-based distance matrix calculator. The NCBI taxonomy is shown to the right of the organism’s name, Figure S2: best-scoring ML phylogenetic tree constructed with 90 amino acid sequences of GlcH protein. Bold numbers to the right of NCBI accession indicate the number of multiple instances of encoded proteins. The numbers near the tree branches indicate the TBE values. The total number of bootstrap trees was 1000. The scale bar shows 0.5 estimated substitutions per site, and *Gloeobacter violaceus* PCC 7421 was used as an outgroup, Figure S3: nucleotide alignment and ABC transporter ATP-binding protein genes of *Planktothrix* phage PaV-LD and *Planktothrix agardhii* NIES-204.
