# Supplementary figures and images for: Tychonema sp. BBK16 Characterisation: Lifestyle, Phylogeny and Related Phages
Source: Viruses. 2023 Feb 5;15(2):442. doi: 10.3390/v15020442 (PMC9958718; doi:10.3390/v15020442)

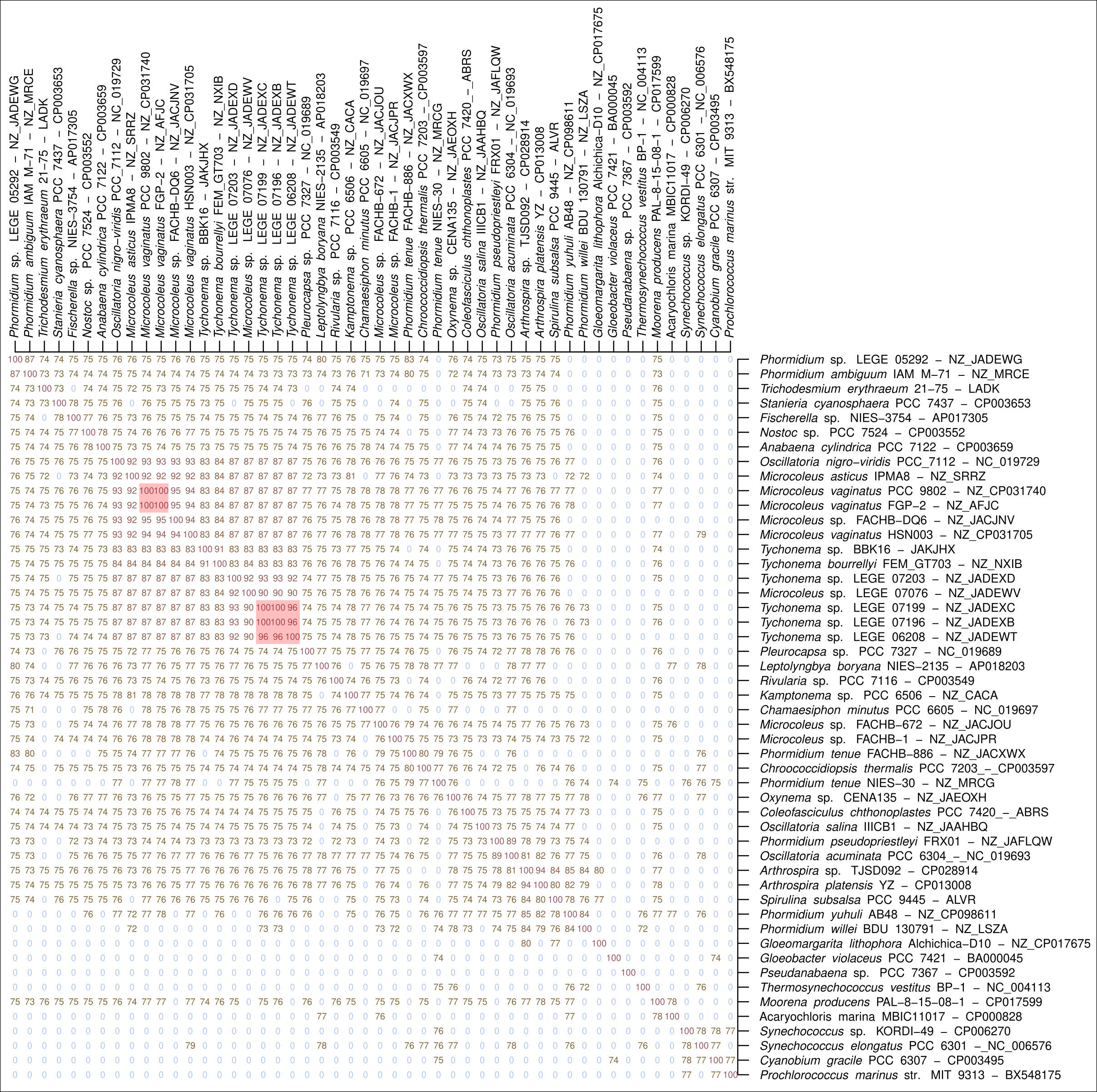

Supplement: Supplementary file 1 [file viruses-15-00442-s001.zip › Supplementary_Figure S1.jpg]

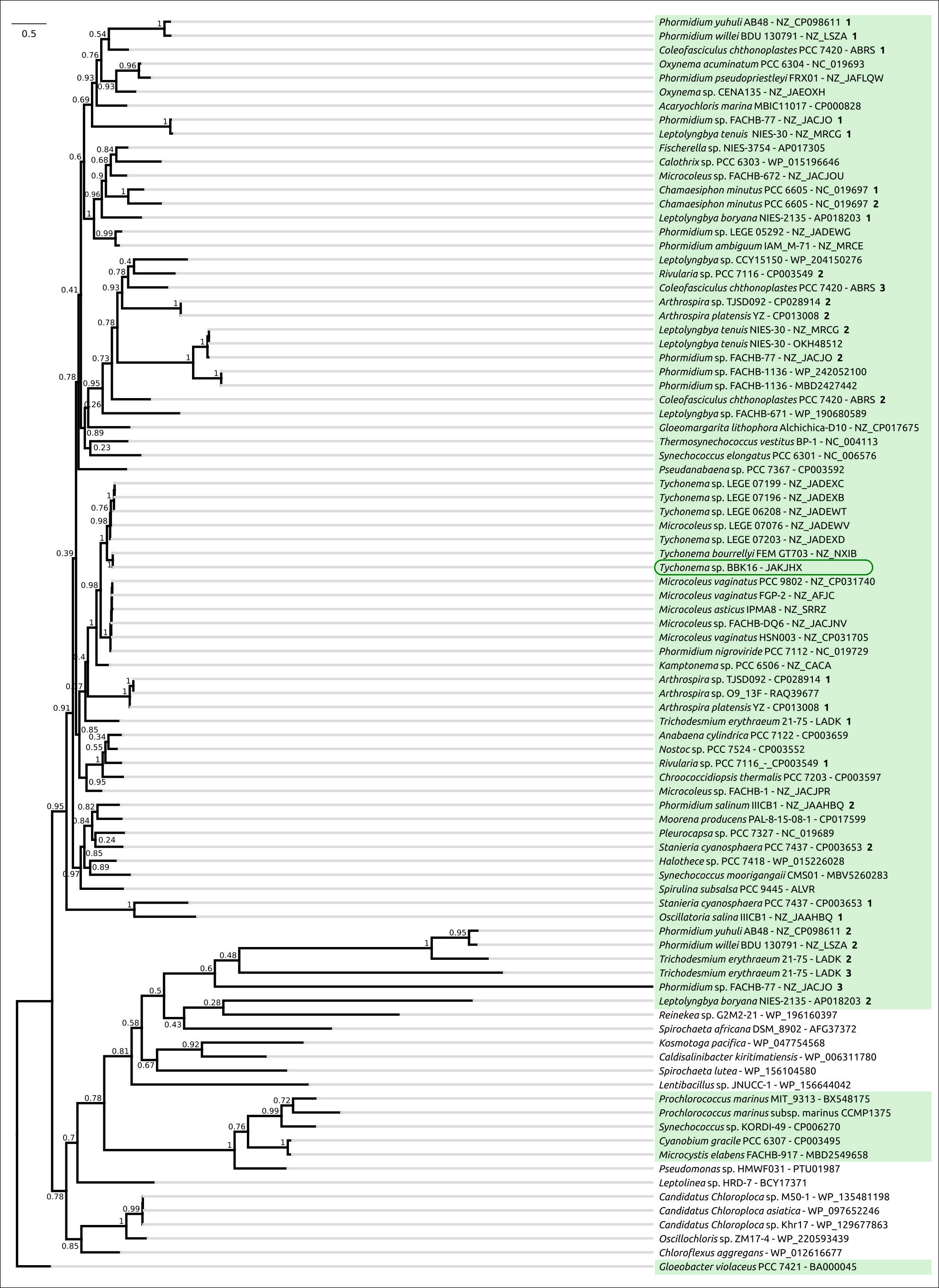

Supplement: Supplementary file 1 [file viruses-15-00442-s001.zip › Supplementary_Figure S2.jpg]

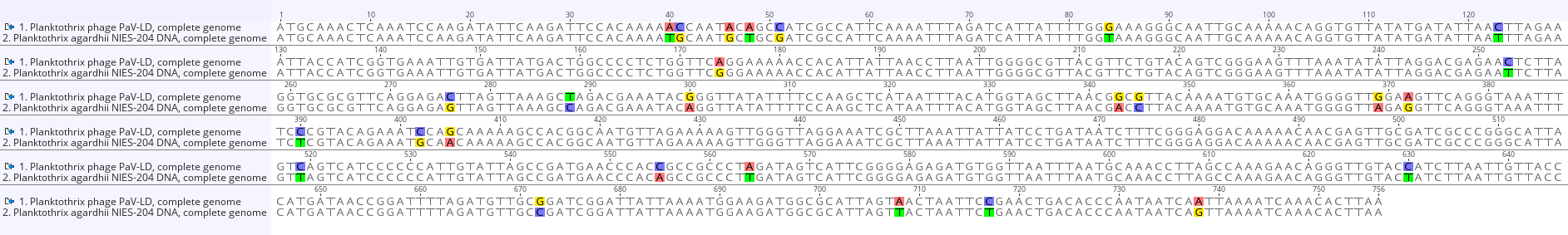

Supplement: Supplementary file 1 [file viruses-15-00442-s001.zip › Supplementary_Figure S3.png]
